# Supplementary material for: Pro-Environmental Behaviors: Determinants and Obstacles among Italian University Students
Source: Int J Environ Res Public Health. 2021 Mar 23;18(6):3306. doi: 10.3390/ijerph18063306 (PMC8004768; doi:10.3390/ijerph18063306)
Supplement: Supplementary file 1 [file ijerph-18-03306-s001.zip › ijerph-1123281-supplementary.pdf]

AmbSal

Questionnaire on environmental and health awareness and behaviour

Dear Students,
Citizens and politicians are interested in environmental pollution due to its multiple effects on the climate, economy, quality of life, and more specifically on health. The public debate on this issue is broad and widespread through many media and information sources.
Citizens have a very important role both in the production of pollution and in its reduction, by their behaviour and through the political pressures they can exert.
This study investigates the sources of information, risk perception, attitudes and behaviour towards environmental pollution, in order to promote information and educational interventions.
To help us, we would therefore like to ask you to answer these questions.

It should take you about 15 minutes to complete the questionnaire.

Thanks for your collaboration!

1. SOCIO-DEMOGRAPHIC CHARACTERISTICS

Gender ☐ **M** ☐ **F**

Age (years) ☐ **Place of residence** ________________**Prov.**______

Degree course ☐ **Bechelor's degree** ☐ **Master's degree**

Sector ☐ **Scientific-Health** ☐ **Humanistic-Legal-Social**

.... In **which city do you live? (may be different from the place of residence)**

How long have you lived there? ☐☐ **Years** ☐☐ **Months**

2. INFORMATION

2.1 Where do you receive most of your information on the relationship between health and environment? (maximum two answers)

☐ Newspapers ☐ Internet ☐ Weekly ☐ Social networks ☐ TV ☐ Radio ☐ Other

2.2 How would you judge this information?

☐ Truthful and complete ☐ Not truthful or complete ☐ Truthful, but incomplete ☐ Don't know

2.3 How do you evaluate your knowledge about the relationship between health and the environment?

☐ Satisfying ☐ Incomplete ☐ Scarce

2.4 How much trust do you have in the information on health risks from the following sources?

1. None **2.** Little **3.** Limited **4.** A lot **5.** I don't use this source of information

	1	2	3	4	5		1	2	3	4	5
**1. TV and radio**						**9. Local community stakeholders**					
**2. Newspapers and magazines**						**10. Municipalities**					
**3. Internet**						**11. Ministry of Environment**					
**4. Social network**						**12. Ministry of Health**					
**5. Friends and relatives**						**13. Regional Environmental Protection Agency**					
**6. Physicians**						**14. Public Health Agencies**					
**7. Alternative medicine experts**						**15. University and Research Institutions**					
**8. Environmentalist Associations**						**16. Industry**					

3. RISK PERCEPTION

3.1 How important is the environment for the development of the following diseases?

1. Not important **2.** Not very important **3.** Quite important **4.** Very important **5.** Extremely important **6**. Don't know

	1	2	3	4	5	6
**1. Tumours**						
**2. Heart diseases**						
**3. Dementia and neurological diseases**						
**4. Congenital malformations**						
**5. Infectious diseases**						
**6. Respiratory diseases**						

3.2 In your opinion, what is the percentage of diseases due to environmental pollution in the world?

☐ 0-20% ☐ 21-40% ☐ 41-60% ☐ 61-80% ☐ > 80% ☐ Don't know

3.3 How important is the health risk to the population resulting from the following?

1.Not important **2.** Not very important **3.** Quite important **4.** Very important **5.** Extremely important **6**. Don't know

	1	2	3	4	5	6		1	2	3	4	5	6
**1. Earthquakes and volcanic eruptions**							**14. Chemicals in food or drinking waters**						
**2. Hydrogeological instability and floods**							**15. Germs in food or drinking waters**						
**3. Climate change**							**16. Food additives**						
**4. Hole in the ozone layer**							**17. Shortage of water**						

**5. Road accidents**							**18. Pollution of groundwater**						
**6. Nuclear facilities**							**19. Pollution of coasts, rivers and lakes**						
**7. Car traffic**							**20. Outdoor air quality**						
**8. Heating systems**							**21. Indoor air quality**						
**9. Industrial discharges and emissions**							**22. Traffic noise**						
**10. Thermoelectric power plants**							**23. Waste and dirt in the streets**						
**11. Accidents in industrial plants**							**24. Landfills**						
**12. High voltage lines, radio and TV repeaters, mobile phones**							**25. Incinerators**						
**13. Genetically modified food (GMOs)**													

3.4 Can you quantify the importance of the health risk arising from the following behaviours?

1. Not important **2.** Not very important **3.** Quite important **4.** Very important **5.** Extremely important **6**. Don't know

	1	2	3	4	5	6
**1. Exposure to sunlight without protection**						
**2. Smoking**						
**3. Use of wood or pellet stoves**						
**4. Improper use of chemicals in the home and in the garden**						
**5. Poor food storage**						

3.5 For each statement, please indicate whether your level of agreement with the following statements

1. Strongly disagree **2.** Disagree **3.** Agree **4.** Strongly agree **5.** Don't know

	1	2	3	4	5
**1. In the place where I live, the environment is a source of health problems**					
**2. I believe my local area is becoming a healthier place to live**					
**3. Soil, air and water are now more polluted than ever**					
**4. I can control my health risks**					
**5. Experts are able to make accurate estimates of health risks from chemicals in the environment**					
**6. I believe I am in good health**					

4. ATTITUDES

4.1 How important are the following subjects in protecting the general population from environmental health hazards?

1. Not important **2.** Not very important **3.** Quite important **4.** Very important **5.** Extremely important **6**. Don't know

	1	2	3	4	5	6		1	2	3	4	5	6
**1. Ministry of Health**							**7. Physicians**						
**2. Public Health Agencies**							**8. "Ecolabel" industries**						
**3. Ministry of Environment**							**9. Environmentalist Associations**						
**4. Regional Environmental Protection Agencies**							**10. Local community stakeholders**						
**5. Municipalities**							**11. Individual citizens**						
**6. Regional governments**							**12. Non-Governmental Organizations**						

4.2 To what extent do the following subjects fulfil in protecting the population from environmental health risks?

1. Scarce **2.** Sufficient **3.** Medium **4.** High **5.** Very high **6.** Don't know

	1	2	3	4	5	6		1	2	3	4	5	6
**1. Ministry of Health**							**7. Physicians**						
**2. Public Health Agencies**							**8. "Ecolabel" industries**						
**3. Ministry of Environment**							**9. Environmentalist Associations**						
**4. Regional Environmental Protection Agencies**							**10. Local community stakeholders**						
**5. Municipalities**							**11. Individual citizens**						
**6. Regional governments**							**12. Non-Governmental Organizations**						

4.3 To what extent do you support the following measures to limit air pollution?

1. Strongly disagree **2.** Disagree **3.** Agree **4.** Strongly agree **5.** Don't know

	1	2	3	4	5
**1. Limitation of vehicular traffic in the city**					
**2. Closure of the center to vehicular traffic**					
**3. Toll parking**					
**4. Alternative transport (cycle paths, public transport development)**					
**5. Temperature limit for domestic heating**					
**6. Decentralization of industries**					

4.4 In your opinion, how important are the following behaviours of citizens in the fight against pollution?

1.Not important **2.** Not very important **3.** Quite important **4.** Very important **5.** Extremely important **6**. Don't know

	1	2	3	4	5	6
**1. Separate collection of waste**						
**2. Use less polluting fuels**						
**3. Buy products with low impact on the environment**						
**4. Reduce energy consumption**						
**5. Buy cars with low emissions**						
**6. Use public transport**						

4.5 Indicate your level of potential support for the following initiatives

1.Very low **2.** Low **3.** Neither high nor low **4.** High **5.** Very high **6.** Don't know

	1	2	3	4	5	6
**1. A new incinerator in your municipality**						
**2. A new landfill in your municipality**						
**3. A new high voltage line within 500 m of your home**						
**4. An underground oil / gas pipeline within 1 km of your home**						
**5. A new highway within 1 km of your home**						
**6. Establishing a natural park around your home**						

5. BEHAVIOURS

5.1 Do you smoke? ☐ Yes ☐ No

If YES: For how many years? |___|___|, how many cigarettes do you smoke per day? |___|___|

(If occasional smoke indicate <1)

If NO: ☐ **How long ago did you stop? |___|___| Years |___|___| Months**

☐ **I have never smoked**

5.2 How often have you adopted the following behaviours?

1. Never **2.** Rarely **3.** Yes, sometimes **4.** Yes, always

	1	2	3	4
**1. Separate collection of waste**				
**2. Use public transport**				
**3. Reduce energy consumption**				
**4. Use less polluting fuels (e.g. methane, electricity)**				
**5. Buy products with low impact on the environment (e.g. zero km, biodegradable)**				

5.3 What obstacles do you find in implementing them? (report obstacles, even more than one, for each behaviour)

BEHAVIOUR \ OBSTACLES	Lack of support from institutions	Lack of support from family / neighbours / acquaintances	Lack of time	Mistrust in effectiveness	Costs
Separate collection of waste					
Use public transport					
Reduce energy consumption					
Use less polluting fuels (e.g. methane, electricity)					
Buy products with low impact on the environment					

TEST FOR FUNCTIONAL HEALTH LITERACY

Lastly, associate the words listed below with the corresponding body part.

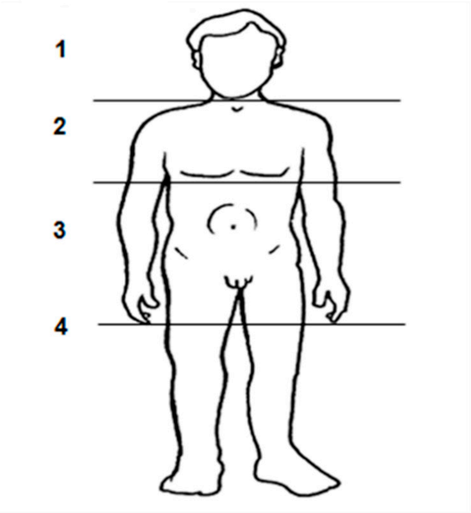

[1] Cold

☐ Headache	☐ Meniscus
☐ Laxative	☐ Rhinitis
☐ Emphysema	☐ Hepatic damage
☐ Oral route	☐ Alveoli
☐ Nephritis	☐ Peptic Ulcer
☐ Constipation	☐ Hematuria
