# Supplementary material for: Pro-Environmental Behaviors: Determinants and Obstacles among Italian University Students
Source: Int J Environ Res Public Health. 2021 Mar 23;18(6):3306. doi: 10.3390/ijerph18063306 (PMC8004768; doi:10.3390/ijerph18063306)
Supplement: Supplementary file 1 [file ijerph-18-03306-s001.zip › Supplemental material - Tables S1 to S8.pdf]

Supplementary Materials

Pro-environmental behaviors: determinants and obstacles among Italian university students

Annalaura Carducci[1], Maria Fiore[2], Antonio Azara[3], Guglielmo Bonaccorsi[4], Martina Bortoletto[5], Giuseppina Caggiano[6], Andrea Calamusa[1], Antonella De Donno[7], Osvalda De Giglio[6], Marco Dettori[3], Pamela Di Giovanni[8], Angela Di Pietro[9], Alessio Facciolà[10], Ileana Federigi[1], Iolanda Grappasonni[11], Alberto Izzotti[12,13], Giovanni Libralato[14], Chiara Lorini[4], Maria Teresa Montagna[6], Liberata Keti Nicolosi[15], Grazia Paladino[15], Giacomo Palomba[1], Fabio Petrelli[11], Tiziana Schilirò[16], Stefania Scuri[11], Francesca Serio[7], Marina Tesauro[17], Marco Verani[1], Marco Vinceti[18,19], Federica Violi[18], Margherita Ferrante[2]*

Affiliations of the Authors:

[1] Department of Biology, University of Pisa, Via S. Zeno 35, 56127 Pisa, Italy
[2] Department of Medical, Surgical Sciences and Advanced Technologies "G. F. Ingrassia", Catania University, Catania, Italy
[3] Department of Medical, Surgical and Experimental Sciences, University of Sassari, via Padre Manzella 4, 07100 Sassari, Italy
[4] Department of Health Science, University of Florence, Viale Morgagni 48, 50134 Florence, Italy.
[5] AZIENDA ULSS 6 EUGANEA, Servizio di Prevenzione, Igiene e Sicurezza negli Ambienti di Lavoro (SPISAL), Via Ospedale n. 22 – 35121 Padova
[6] Department of Biomedical Sciences and Human Oncology, University of Bari "Aldo Moro", Piazza G. Cesare 11, 70124 Bari, Italy
[7] Laboratory of Hygiene, Department of Biological and Environmental Sciences and Technology, University of Salento, 73100 Lecce, Italy
[8] Department of Pharmacy, "G. d'Annunzio" University of Chieti-Pescara, via dei Vestini 31, 66100 Chieti, Italy
[9] Department of Biomedical and Dental Sciences and Morphofunctional Imaging, University of Messina
[10] Department of Clinical and Experimental Medicine, Unit of Infectious Diseases, University of Messina
[11] University of Camerino, School of Medicinal and Health Products Sciences, Via Madonna delle Carceri 9, 62032 Camerino, Italy
[12] Department of Experimental Medicine, School of Medicine, University of Genoa, Italy.
[13] IRCCS Ospedale Policlinico San Martino Genova, Italy
[14] Department of Biology, University of Naples Federico II, via Cinthia 21, 80126 Naples, Italy
[15] Specialization School of Hygiene and Preventive Medicine. Department of Medical, Surgical Sciences and Advanced Technologies "G.F. Ingrassia", Catania University, Catania, Italy
[16] Department of Public Health and Pediatrics, University of Torino, Piazza Polonia 94, 10126 Torino, Italy
[17] Department of Biomedical, Surgical and Dental Sciences, University of Milan, Italy
[18] Section of Public Health, Department of Biomedical, Metabolic and Neural Sciences, University of Modena and Reggio Emilia, Via Campi 287, 41125 Modena, Italy.
[19] Department of Epidemiology, Boston University School of Public Health, Boston, Massachusetts (USA)

* **Corresponding author**: Ileana Federigi. Department of Biology, University of Pisa, Via S. Zeno 35/39, Pisa 56127, Italy – ileana.federigi@biologia.unipi.it

Table S1. Items (and related question) used for the calculation of the global indexes.

Global indexes	Question	Items used for global index's calculation
**Global indexes calculated in the present work**		
Global Negative Attitudes index (GNA)	Indicate your level of potential support for the following initiatives	− A new incinerator in your municipality<br>− A new landfill in your municipality<br>− A new high voltage line within 500 m of your home<br>− An underground oil / gas pipeline within 1 km of your home<br>− A new highway within 1 km of your home
Global Support of measures against air pollution (GS)	To what extent do you support the following measures to limit air pollution?	− Limitation of vehicular traffic in the city<br>− Closure of the center to vehicular traffic<br>− Toll parking<br>− Alternative transport (cycle paths, public transport development)<br>− Temperature limit for domestic heating<br>− Decentralization of industries
Positive attitudes for pro-environmental behaviors (PAPEB)	In your opinion, how important are the following behaviours of citizens in the fight against pollution?	− Separate collection of waste<br>− Use less polluting fuels<br>− Buy products with low impact on the environment<br>− Reduce energy consumption<br>− Buy cars with low emissions<br>− Use public transport
Adoption of pro-environmental behaviors (APEB)	How often have you adopted the following behaviours?	− Separate collection of waste<br>− Use public transport<br>− Reduce energy consumption<br>− Use less polluting fuels (e.g. methane, electricity)<br>− Buy products with low impact on the environment (e.g. zero km, biodegradable)
**Global indexes coming from previous analysis**		
Global Health Risk Perception (GHRP)	How important is the health risk to the population resulting from the following?	− Earthquakes and volcanic eruptions<br>− Hydrogeological instability and floods<br>− Climate change<br>− Hole in the ozone layer<br>− Road accidents<br>− Nuclear facilities<br>− Car traffic<br>− Heating systems<br>− Industrial discharges and emissions<br>− Thermoelectric power plants

		− Accidents in industrial plants<br>− High voltage lines, radio and TV repeaters, mobile phones<br>− Genetically modified food (GMOs)<br>− Chemicals in food or drinking water<br>− Germs in food or drinking waters<br>− Food additives<br>− Shortage of water<br>− Pollution of groundwater<br>− Pollution of coasts, rivers and lakes<br>− Outdoor air quality<br>− Indoor air quality<br>− Traffic noise<br>− Waste and dirt in the streets<br>− Landfills<br>− Incinerators
Trust in action by institutional subjects (TAI)	To what extent do the following subjects fulfil in protecting the population from environmental health risks?	− Ministry of Health<br>− Public Health Agencies<br>− Ministry of Environment<br>− Regional Environmental Protection Agencies<br>− Municipalities<br>− Regional governments<br>− Physicians
Trust in action by non-institutional subjects (TANI)		− "Ecolabel" industries<br>− Environmentalist Associations<br>− Local community stakeholders<br>− Individual citizens<br>− Non-Governmental Organizations

Table S2. Associations between negative attitudes towards interventions (single items and global index (GNA)) and gender, area of residence, functional health literacy, global health risk perception index, trust in institution index, internal locus of control, and sources of information. Notable associations between negative attitudes and the other variables are highlighted by bold Spearman's rho coefficient.

	**Gender** (Mean ± SD)	**Area of Residence** (Mean ± SD)	**Functional health literacy** (Mean ± SD)	**Global health risk perception** (Rho, 95% CI)	**Trust in institution fulfillment** (Rho, 95% CI)	**Internal Locus of Control** (Rho, 95% CI)	**Sources of information** (Mean ± SD)
A new landfill in your municipality	F = 1.9±1.3<br>M = 1.8±1.2	North = 1.9±1.3<br>Centre = 1.8±1.2<br>South = 1.9±1.3	>9 = 1.8±1.2<br><9 = 1.9±1.3	Rho = -0.019<br>95%CI: -0.047 – 0.009	**Rho = 0.17**<br>95%CI: 0.143 – 0.197	Rho = -0,033<br>95%CI: -0.061 – -0.005	IS = 1.9±1.3<br>DW = 1.9±1.3<br>O = 1.9±1.4
An underground oil/gas pipeline within 1 km from your home	F = 1.7±1.2<br>M = 1.7±1.2	North = 1.8±1.2<br>Centre = 1.7±1.2<br>South = 1.7±1.2	>9 = 1.7±1.2<br><9 = 1.7±1.3	Rho = -0.038<br>95%CI: -0.066 – -0.01	**Rho = 0.172**<br>95%CI:0.145-0.199	Rho = -0.058<br>95%CI: -0.086 – -0.03	IS =1.7 ±1.2<br>DW = 1.8±1.3<br>O = 1.7±1.3
A new highway within 1 km of your home	F = 1.9±1.2<br>M = 1.9±1.2	North = 1.9±1.2<br>Centre = 1.8±1.2<br>South = 1.9±1.2	>9 = 1.9±1.2<br><9 = 1.9±1.2	Rho = -0.053<br>95%CI: -0.081 – -0.25	**Rho = 0.122**<br>95%CI:0.094 – 0.150	Rho = -0.063<br>95%CI: -0.091 – -0.035	IS = 1.9 ±1.2<br>DW = 1.8±1.2<br>O = 1.9±1.3
A new incinerator in your municipality	F = 1.8±1.2<br>M =1.8 ±1.2	North= 1.9±1.3<br>Centre= 1.8±1.3<br>South= 1.8±1.3	>9 = 1.8±1.3<br><9 = 1.8±1.3	Rho = 0.006<br>95%CI: -0.022 – 0.034	**Rho=0.185**<br>**p=0.000**<br>95%CI: 0.158 – 0.212	Rho = -0.011<br>95%CI: -0.039 – 0.017	IS = 1.8±1.3<br>DW = 1.8±1.3<br>O = 1.8±1.3
A new high voltage line within 500 m of your home	F = 1.8±1.2<br>M = 1.7±1.2	North = 1.7±1.2<br>Centre = 1.7±1.2<br>South = 1.8±1.2	>9 =1.7 ±1.2<br><9 = 1.8±1.3	Rho = -0.027<br>95%CI: -0.055 – 0.001	**Rho = 0.168**<br>95%CI: 0.141 – 0.191	Rho = -0.032<br>95%CI: -0.06 – -0.004	IS = 1.7±1.2<br>DW =1.7 ±1.2<br>O = 1.8±1.3
Global Negative Attitudes (GNA) index	F = 12.9±5.1<br>M = 13.2±4.9	North = 13.4±5.8<br>Centre = 12.9±4.8<br>South = 13.1±5.0	>9 = 13.1±4.9<br><9 = 13.2±5.2	Rho = -0.015<br>95%CI: -0.043 – 0.013	**Rho = 0.196**<br>95%CI: 0.169 – 0.223	Rho = -0.025<br>95%CI: -0.053 – 0.003	IS = 13.1±5.0<br>DW =13.2 ±5.0<br>O = 12.8±5.5

IS: Internet and Social declared as sources of information (even not exclusive)
DW: Daily and Weekly newspapers declared a sources of information (even not exclusive)
O: Other sources of information.

Table S3. Associations between level of support of measures against air pollution (single items and global index (GS)) and gender, area of residence, functional health literacy, global health risk perception index, trust in institution index, internal locus of control, and sources of information. Notable associations between level of support of measures against air pollution and the other variables are highlighted by bold Spearman's rho coefficient.

	**Gender** Mean ± SD	**Area of Residence** (Mean ± SD)	**Functional health literacy** (Mean ± SD)	**Global health risk perception** (Rho, 95% CI)	**Trust in institution fulfillment** (Rho, 95% CI)	**Internal Locus of Control** (Rho, 95% CI)	**Sources of information** (Mean ± SD)
Alternative transport	F = 3.4±1.0<br>M = 3.5±1.0	North = 3.5±1.0<br>Centre = 3.5±1.1<br>South = 3.4±1.0	>9 = 3.5±0.9<br><9 = 3.3±1.1	Rho = 0.059<br>95% CI:0.031 – 0.087	Rho = -0.009<br>95% CI: -0.037 – 0.019	**Rho = 0.164**<br>95% CI: 0.137 – 0.191	IS = 3.46±1.0<br>DW = 3.46±1.0<br>O =3.33 ±1.1
Limitation of vehicular traffic	F = 3.2±1.0<br>M = 3.2±1.0	North = 3.2±1.0<br>Centre = 3.2±1.0<br>South = 3.2±1.0	>9 =3.3 ±0.9<br><9 = 3.1±1.1	**Rho = 0.147**<br>95% CI: 0.120 – 0.174	Rho = 0.034<br>95% CI: 0.006 – 0.062	**Rho = 0.157**<br>95% CI:0.130 – 0.184	IS = 3.23±1.0<br>DW = 3.22±1.0<br>O = 3.12±1.0
Decentralization of industries	F = 3.0±1.3<br>M = 2.9±1.4	North= 3.0±1.4<br>Centre= 2.9±1.4<br>South= 3.0±1.3	>9 = 3.0±1.3<br><9 = 2.9±1.4	Rho = 0.057<br>95% CI: 0.029 – 0.085	Rho = -0.010<br>95% CI: -0.038 – 0.018	**Rho = 0.112**<br>95% CI: 0.084 – 0.139	IS = 2.97±1.3<br>DW = 2.91±1.4<br>O = 2.82±1.4
Closure of the city center to vehicular traffic	F = 2.9±1.1<br>M = 2.9±1.1	North = 2.9±1.1<br>Centre = 3.0±1.1<br>South = 2.9±1.1	>9 = 3.0±1.0<br><9 = 2.9±1.1	**Rho = 0.104**<br>95% CI: 0.076 -0.131	Rho = 0.048<br>95% CI: 0.020 – 0.076	**Rho = 0.117**<br>95% CI: 0.089 – 0.144	IS = 2.93 ±1.1<br>DW = 2.92±1.2<br>O = 2.84±1.0
Temperature limit for domestic heating	F = 2.7±1.2<br>M = 2.7±1.2	North = 2.7±1.2<br>Centre = 2.8±1.2<br>South = 2.7±1.2	>9 = 2.8±1.1<br><9 = 2.6±1.3	Rho=0.040<br>95% CI: 0.012 -0.068	Rho = -0.014<br>95% CI: -0.042 – 0.014	**Rho = 0.138**<br>95% CI: 0.111-0.165	IS = 2.72±1.2<br>DW = 2.84±1.2<br>O = 2.61±1.2
Toll parking	F = 1.9±1.1<br>M = 1.8 ±1.1	North = 1.9±1.1<br>Centre = 1.9±1.1<br>South = 1.9±1.0	>9 = 1.9±1.0<br><9 = 1.9±1.1	Rho = 0.029<br>95% CI: 0.001-0.057	Rho=0.049<br>95% CI: 0.021 – 0.077	Rho = 0.078<br>95% CI: 0.050 - 0.106	IS = 1.86±1.1<br>DW = 1.91±1.2<br>O = 1.77±1.0
Global Support (GS) Index	F = 17.1±4.2<br>M = 17.1±4.1	North = 17.2±4.0<br>Centre = 17.2±4.4<br>South = 17.1±4.1	>9 = 17.5±3.8<br><9 = 16.7±4.6	**Rho = 0.267**<br>95% CI: 0.241 – 0.293	**Rho = 0.137**<br>95% CI: 0.110 – 0.164	**Rho = 0.185**<br>95% CI: 0.158 – 0.212	IS = 17.2±4.1<br>DW = 17.3±4.3<br>O = 16.5±4.3

IS: Internet and Social declared as sources of information (even not exclusive)
DW: Daily and Weekly newspapers declared a sources of information (even not exclusive)
O: Other sources of information.

Table S4. Associations between positive attitudes for pro-environmental behaviors (single items and global index (PAPEB)) and gender, area of residence, functional health literacy, global health risk perception index, trust in institution index, internal locus of control, and sources of information. Notable associations between positive attitudes and the other variables are highlighted by bold Spearman's rho coefficient.

	**Gender** Mean ± SD	**Area of Residence** (Mean ± SD)	**Functional health literacy** (Mean ± SD)	**Global health risk perception** (Rho, 95% CI)	**Trust in institution fulfillment** (Rho, 95% CI)	**Internal Locus of Control** (Rho, 95% CI)	**Sources of information** (Mean ± SD)
Separate collection of waste	F = 4.3±1.1<br>M= 4.3±1.1	North = 4.4±1.0<br>Centre = 4.3±1.2<br>South = 4.3±1.1	>9 = 4.4±0.9<br><9 = 4.2±1.2	**Rho = 0.274**<br>95% CI: 0.248 – 0.300	**Rho = 0.112**<br>95% CI: 0.084 – 0.139	**Rho = 0.199**<br>95% CI: 0.172 – 0.226	IS = 4.31±1.1<br>DW = 4.29±1.2<br>O = 4.25±1.1
Use of less polluting fuels	F = 4.3±1.1<br>M= 4.3±1.1	North = 4.4±1.0<br>Centre = 4.3±1.1<br>South = 4.3±1.0	>9 = 4.4±0.9<br><9 = 4.2±1.2	**Rho = 0.336**<br>95% CI: 0.311 – 0.360	Rho = 0.063<br>95% CI:0.035 – 0.091	**Rho = 0.218**<br>95% CI: 0.191 – 0.244	IS = 4.32±1.0<br>DW = 4.31±1.1<br>O = 4.21±1.1
Buy products with low impact on the environment	F = 4.1±1.1<br>M= 4.2±1.1	North = 4.3 ±1.0<br>Centre = 4.1±1.2<br>South = 4.1±1.1	>9 = 4.2±1.0<br><9 = 4.1±1.2	**Rho = 0.349**<br>95% CI: 0.324 – 0.373	Rho = 0.074<br>95% CI: 0.046 – 0.102	**Rho = 0.225**<br>95% CI: 0.198 – 0.251	IS = 4.15±1.1<br>DW = 4.15±1.2<br>O = 4.08±1.2
Buy cars with low emissions	F = 4.2±1.1<br>M= 4.1 ±1.1	North = 4.2±1.0<br>Centre = 4.1±1.2<br>South = 4.1±1.1	>9 = 4.2±1.0<br><9 = 4.0±1.2	**Rho = 0.305**<br>95% CI: 0.280 – 0.330	Rho = 0.080<br>95% CI: 0.052 – 0.108	**Rho = 0.211**<br>95% CI: 0.184 – 0.237	IS = 4.16±1.1<br>DW = 4.10±1.2<br>O = 4.06±1.2
Reduce energy consumption	F = 4.1±1.1<br>M= 4.2±1.1	North = 4.2±1.0<br>Centre = 4.1±1.2<br>South = 4.2±1.1	>9 = 4.2±1.0<br><9 = 4.1±1.2	**Rho = 0.283**<br>95% CI: 0.257 – 0.308	Rho = 0.071<br>95% CI: 0.043 – 0.099	**Rho = 0.208**<br>95% CI: 0.181 – 0.234	IS = 4.16±1.1<br>DW = 4.15±1.2<br>O = 4.10±1.1
Use of public transport	F = 4.0±1.3<br>M = 3.9±1.3	North = 4.0±1.3<br>Centre = 3.9±1.4<br>South = 3.9±1.3	>9 = 4.1±1.2<br><9 = 3.8±1.4	**Rho = 0.190**<br>95% CI: 0.163 – 0.217	Rho = 0.040<br>95% CI: 0.012 – 0.068	**Rho = 0.207**<br>95% CI: 0.180 – 0.233	IS = 3.95±1.3<br>DW = 3.91±1.4<br>O = 3.85±1.3
Global Positive Attitudes for Pro Environmental Behaviors (PAPEB) index	F = 25.0 ± 5.4<br>M = 24.9 ± 5.3	North = 25.4±4.9<br>Centre = 24.8±5.8<br>South = 25.0±5.3	>9 = 25.5±4.7<br><9 = 24.3±6.0	**Rho = 0.376**<br>95% CI: 0.180 – 0.233	**Rho = 0.113**<br>95% CI: 0.085 – 0.140	**Rho = 0.283**<br>95% CI: 0.257 – 0.308	IS = 25.1±5.2<br>DW = 24.9±5.9<br>O = 24.5±5.6

IS: Internet and Social declared as sources of information (even not exclusive)
DW: Daily and Weekly newspapers declared a sources of information (even not exclusive)
O: Other sources of information.

Table S5. Associations between adoption of pro-environmental behaviors (single items and global index (APEB)) and gender, area of residence, functional health literacy, global health risk perception index, trust in institution index, internal locus of control, and sources of information. Notable associations between pro-environmental behaviors and the other variables are highlighted by bold Spearman's rho coefficient.

	**Gender** (Mean ± SD)	**Area of Residence** (Mean ± SD)	**Functional health literacy** (Mean ± SD)	**Global health risk perception** (Rho, 95% CI)	**Trust in institution fulfillment** (Rho, 95% CI)	**Internal Locus of Control** (Rho, 95% CI)	**Sources of information** (Mean ± SD)
Separate collection of waste	F = 3.5±0.8<br>M = 3.5±0.8	North = 3.5±0.8<br>Centre = 3.6±0.7<br>South = 3.5±0.8	>9 = 3.5±0.8<br><9 = 3.5±0.8	Rho = 0.077<br>95% CI: 0.049 – 0.105	Rho = 0.005<br>95% CI: -0.023 – 0.033	Rho = 0.073<br>95% CI: 0.045 – 0.101	IS = 3.50±0.8<br>DW = 3.59±0.7<br>O = 3.41±0.9
Use of less polluting fuels	F = 2.7±0.9<br>M = 2.7±0.9	North =2.7 0.9±<br>Centre = 2.7±0.9<br>South = 2.6±0.9	>9 = 2.7±0.9<br><9 = 2.6±0.9	**Rho = 0.133**<br>95% CI: 0.105 – 0.160	Rho = 0.037<br>95% CI:0.009 – 0.065	Rho = 0.074<br>95% CI: 0.046 – 0.102	IS = 2.65±0.9<br>DW = 2.77±0.9<br>O = 2.56±0.9
Buy products with low impact on the environment	F = 2.7±0.8<br>M = 2.8±0.8	North = 2.8±0.8<br>Centre = 2.8±0.8<br>South = 2.7±0.8	>9 =2.8±0.8<br><9 =2.7±0.8	**Rho = 0.125**<br>95% CI: 0.097 – 0.153	Rho = 0.041<br>95% CI:0.013 – 0.069	Rho = 0.096<br>95% CI: 0.068 – 0.124	IS = 2.73 ±0.8<br>DW = 2.85 ±0.8<br>O = 2.64±0.8
Reduce energy consumption	F = 2.9 ±0.8<br>M = 2.9 ±0.7	North = 2.9±0.8<br>Centre = 2.9±0.8<br>South = 2.9±0.8	>9 = 2.9±0.7<br><9 = 2.9±0.8	**Rho = 0.136**<br>95% CI: 0.108 – 0.163	Rho = 0.059<br>95% CI:0.031 – 0.087	Rho = 0.067<br>95% CI: 0.039 – 0.095	IS = 2.88±0.8<br>DW = 2.99±0.8<br>O = 2.85±0.8
Use of public transport	F = 2.9±0.8<br>M = 2.9±0.8	North = 2.9±0.8<br>Centre = 2.9±0.8<br>South = 2.9±0.8	>9 = 2.9±0.8<br><9 = 3.0±0.8	Rho = -0.016<br>95% CI: -0.012 – 0.044	Rho = 0.027<br>95% CI: -0.001 – 0.055	Rho = 0.010<br>95% CI: -0.018 – 0.038	IS = 2.93 ±0.8<br>DW = 2.97±0.8<br>O = 2.86±0.8
Global Adoption of Pro-Environmental Behaviors (APEB) index	F = 14.7±2.5<br>M = 14.8±2.5	North = 14.8±2.3<br>Centre = 14.9±2.4<br>South = 14.6±2.5	>9 = 14.8±2.5<br><9 = 14.7±2.5	**Rho = 0.154**<br>95% CI: 0.126 – 0.181	Rho = 0.070<br>95% CI: 0.042 – 0.098	**Rho=0.114**<br>95% CI: 0.086 – 0.142	IS = 14.7 ±2.5<br>DW = 15.2±2.4<br>O = 14.3±2.5

IS: Internet and Social declared as sources of information (even not exclusive)
DW: Daily and Weekly newspapers declared a sources of information (even not exclusive)
O: Other sources of information.

Table S6. Association between declared obstacles against pro-environmental behaviors and the following dichotomized variables: gender, area of residence, functional health literacy, health risk perception, trust in institution fulfillment, internal locus of control, internet and social as sources of information. Notable differences are highlighted on a grey background.

	Gender n (%)	Area of Residence	Functional Health literacy	Global Health Risk perception	Trust in instittition fulfillemnt	Internal Locus of control	Internet and social
**SEPARATE COLLECTION OF WASTE**	Cramer'V= 0.030	Cramer'V= 0.074	Cramer'V=0.038	Cramer'V= 0.034	Cramer'V=0.038	Cramer'V=0.018	Cramer'V=0.065
Lack of support from family / neighbours / acquaintances	M= 275 (19.5)<br>F= 518 (19.4)	Centre-north= 363 (19.1)<br>South-islands= 431 (19.8)	High (>9)= 436 (19.3)<br>Low (≤9)= 358 (19.7)	High >75=693 (19.4)<br>Low ≤75= 101 (20.0)	High >21=230(18.2)<br>Low ≤21= 564(20.0)	High >4= 282(18.6)<br>Low ≤4= 512(20.0)	Yes=660 (20.1)<br>No=133(16.6)
Lack of support from institutions	M= 665 (47.2)<br>F= 1219 (45.6)	Centre-north= 911 (47.9)<br>South-islands= 973 (44.6)	High (>9)= 1033 (45.6)<br>Low (≤9)= 851 (46.8)	High >75= 1672 (46.7)<br>Low ≤75 = 2012 (42.0)	High >21=592(46.9)<br>Low ≤21=1292(45.8)	High >4= 704(46.4)<br>Low ≤4=1180(46.0)	Yes=1487(45.4)<br>No=397(49.4)
Costs	M= 39 (2.8)<br>F= 59 (2.2)	Centre-north= 38 (2.0)<br>South-islands= 60 (2.7)	High (>9)= 45 (2.0)<br>Low (≤9)= 53 (2.9)	High >75= 83 (2.3)<br>Low ≤75= 15 (3.0)	High >21=39(3.1)<br>Low ≤21=59(2.1)	High >4= 38(2.5)<br>Low ≤4=60(2.3)	Yes=69(2.1)<br>No=29(3.6)
Lack of time	M= 187 (13.3)<br>F= 360 (13.5)	Centre-north= 211 (11.1)<br>South-islands= 337 (15.4)	High (>9)= 317 (14.0)<br>Low (≤9)=231 (12.7)	High >75 = 476 (13.3)<br>Low ≤75 = 72 (14.3)	High >21=174(13.8)<br>Low ≤21=374(13.3)	High >4= 208(13.7)<br>Low ≤4=340(13.3)	Yes=431(13.1)<br>No=116(14.4)
Mistrust in effectiveness	M= 244 (17.3)<br>F= 515 (19.3)	Centre-north= 378 (19.9)<br>South-islands= 381 (17.5)	High (>9)= 433 (19.1)<br>Low (≤9)= 326 (17.9)	High >75= 654 (18.3)<br>Low ≤75= 105 (20.8)	High >21=227(18.0)<br>Low ≤21=532(18.9)	High >4= 286(18.8)<br>Low ≤4=473(18.4)	Yes=631(19.2)<br>No=128(15.9)
**USE PUBLIC TRANSPORT**	Cramer'V= 0.024	Cramer'V= 0.068	Cramer'V=0.034	Cramer'V=0.028	Cramer'V=0.028	Cramer'V=0.018	Cramer'V=0.029
Lack of support from family / neighbours / acquaintances	M= 55 (3.6)<br>F= 107 (3.8)	Centre-north= 72 (3.6)<br>South-islands= 90 (3.8)	High (>9)=95 (3.9)<br>Low (≤9)=67 (3.5)	High >75= 146 (3.8)<br>Low ≤75= 16 (3.0)	High >21=55(4.1)<br>Low ≤21=107(3.5)	High >4= 55(3.4)<br>Low ≤4=107(3.9)	Yes=129(3.7)<br>No=33(3.8)
Lack of support from institutions	M= 701 (46.2)<br>F= 1329 (46.9)	Centre-north= 983 (49.6)<br>South-islands= 1048 (44.1)	High (>9)= 1155 (47.4)<br>Low (≤9)= 876 (45.6)	High >75 = 1792 (46.9)<br>Low ≤75 =239 (45.0)	High >21=640(47.8)<br>Low ≤21=1391(46.1)	High >4= 761(46.7)<br>Low ≤4=1270(46.6)	Yes=1609(46.2)<br>No=422(48.3)
Costs	M= 239 (15.8)<br>F= 480 (16.9)	Centre-north= 336 (17.0)<br>South-islands= 384 (16.2)	High (>9)= 388 (15.9)<br>Low (≤9)= 332 (17.3)	High >75= 637 (16.7)<br>Low ≤75= 83 (15.6)	High >21=218(16.3)<br>Low ≤21=502(16.6)	High >4= 278(17.1)<br>Low ≤4=442(16.2)	Yes=567(16.3)<br>No=153(17.5)
Lack of time	M= 263 (17.3)<br>F= 449 (15.8)	Centre-north=285 (14.4)<br>South-islands=427 (18.0)	High (>9) =408 (16.8)<br>Low (≤9)= 304 (15.8)	High >75=618 (16.2)<br>Low ≤75=94 (17.7)	High >21=218(16.3)<br>Low ≤21=494(16.4)	High >4= 262(16.1)<br>Low ≤4=450(16.5)	Yes=580(16.7)<br>No=131(15.0)
Mistrust in effectiveness	M= 259 (17.1)<br>F= 469 (16.5)	Centre-north= 304 (15.4)<br>South-islands= 425 (17.9)	High (>9)= 389 (16.0)<br>Low (≤9)= 340 (17.7)	High >75=630 (16.5)<br>Low ≤75=99 (18.6)	High >21=207(15.5)<br>Low ≤21=522(17.3)	High >4= 272(16.7)<br>Low ≤4=457(16.8)	Yes=594(17.1)<br>No=135(15.4)

Table S6. continued

	Gender<br>n (%)	Area of Residence	Functional Health literacy	Global Health Risk perception	Trust in institition fulfillemnt	Internal Locus of control	Internet and social
**REDUCE ENERGY CONSUMPTION**	Cramer'V= 0.051	Cramer'V= 0.344	Cramer'V= 0.036	Cramer'V=0.050	Cramer'V=0.045	Cramer'V=0.021	Cramer'V=0.096
Lack of support from family / neighbours / acquaintances	M= 363 (27.0)<br>F= 690 (26.6)	Centre-north= 410 (21.3)<br>South-islands= 645 (32.0)	High (>9)=588 (26.9)<br>Low (≤9)=467 (26.5)	High >75=904 (26.3)<br>Low ≤75=151 (29.9)	High >21=332(27.6)<br>Low ≤21=723(26.3)	High >4= 402(27.4)<br>Low ≤4=653(26.4)	Yes=903(28.1)<br>No=151(20.8)
Lack of support from institutions	M= 537 (39.9)<br>F= 1064 (41.0)	Centre-north= 1099 (57.0)<br>South-islands= 503 (24.9)	High (>9)= 866 (39.6)<br>Low (≤9)=736 (41.8)	High >75=1404 (40.8)<br>Low ≤75=198 (39.2)	High >21=510(42.5)<br>Low ≤21=1092(39.8)	High >4= 584(39.8)<br>Low ≤4=1018(41.1)	Yes=1275(39.6)<br>No=327(45.0)
Costs	M= 129 (9.6)<br>F= 315 (12.1)	Centre-north= 98 (5.1)<br>South-islands= 346 (17.1)	High (>9) = 244 (11.2)<br>Low (≤9) = 200 (11.4)	High >75=377(11.0)<br>Low ≤75=67(13.3)	High >21=133(11.1)<br>Low ≤21=311(11.3)	High >4= 160(10.9)<br>Low ≤4= 284(11.5)	Yes=371(11.5)<br>No=73(10.1)
Lack of time	M= 168 (12.5)<br>F= 297 (11.4)	Centre-north= 195 (10.1)<br>South-islands= 270 (13.4)	High (>9) = 279 (12.8)<br>Low (≤9) = 186 (10.6)	High >75=410(11.9)<br>Low ≤75=55(10.9)	High >21=119(9.9)<br>Low ≤21=346(12.6)	High >4= 174(11.8)<br>Low ≤4=291(11.7)	Yes=346(10.7)<br>No=119(16.4)
Mistrust in effectiveness	M= 148 (11.0)<br>F= 232 (8.9)	Centre-north= 126 (6.5)<br>South-islands= 254 (12.6)	High (>9) = 210 (9.6)<br>Low (≤9)= 170 (9.7)	High >75=346(10.1)<br>Low ≤75=34(6.7)	High >21=107(8.9)<br>Low ≤21=273(9.9)	High >4= 149(10.1)<br>Low ≤4=231(9.3)	Yes=324(10.1)<br>No=56(7.7)
**USE OF LESS POLLUTING FUELS**	Cramer'V= 0.049	Cramer'V= 0.406	Cramer'V= 0.046	Cramer'V=0.056	Cramer'V=0.017	Cramer'V=0.034	Cramer'V=0.189
Lack of support from family / neighbours / acquaintances	M= 581 (39.6)<br>F= 1136 (42.3)	Centre-north= 129 (6.4)<br>South-islands= 432 (20.1)	High (>9)= 289 (12.4)<br>Low (≤9)= 272 (15.0)	High >75=473(13.0)<br>Low ≤75=88(17.2)	High >21=177(13.9)<br>Low ≤21=384(13.3)	High >4= 200(12.7)<br>Low ≤4=361(14.0)	Yes=486(14.2)<br>No=75(10.3)
Lack of support from institutions	M= 219 (14.9)<br>F= 341 (12.7)	Centre-north= 1240 (61.7)<br>South-islands= 479 (22.3)	High (>9)= 981 (42.0)<br>Low (≤9)= 738 (40.6)	High >75=1521(41.8)<br>Low ≤75=198(38.6)	High >21=522(41.1)<br>Low ≤21=1197(41.5)	High >4= 677(43.1)<br>Low ≤4=1042(40.3)	Yes=1362(39.8)<br>No=356(48.8)
Costs	M= 461 (31.4)<br>F= 794 (29.6)	Centre-north= 429 (21.3)<br>South-islands= 826 (38.5)	High (>9)= 718 (30.7)<br>Low (≤9)= 537 (29.6)	High >75=1089(29.9)<br>Low ≤75=166(32.4)	High >21=377(29.7)<br>Low ≤21=878(30.4)	High >4= 457(29.1)<br>Low ≤4=798(30.9)	Yes=1118(32.7)<br>No=136(18.6)
Lack of time	M= 94 (6.4)<br>F= 218 (8.1)	Centre-north= 107 (5.3)<br>South-islands= 205 (9.6)	High (>9) = 186 (8.0)<br>Low (≤9)= 126 (6.9)	High >75=276(7.6)<br>Low ≤75=36(7.0)	High >21=93(7.3)<br>Low ≤21=219(7.6)	High >4= 113(7.2)<br>Low ≤4=199(7.7)	Yes=194(5.7)<br>No=118(16.2)
Mistrust in effectiveness	M= 112 (7.6)<br>F= 196 (7.3)	Centre-north= 105 (5.2)<br>South-islands= 203 (9.5)	High (>9)= 164 (7.0)<br>Low (≤9)= 144 (7.9)	High >75=283(7.8)<br>Low ≤75=25(4.9)	High >21=101(8.0)<br>Low ≤21=207(7.2)	High >4= 122(7.8)<br>Low ≤4=186(7.2)	Yes=263(7.7)<br>No=45(6.2)
**BUY PRODUCTS WITH LOW IMPACT ON THE ENVIRONMENT**	Cramer'V= 0.047	Cramer'V= 0.546	Cramer'V= 0.013	Cramer'V=0.035	Cramer'V=0.015	Cramer'V=0.055	Cramer'V=0.750
Lack of support from family / neighbours / acquaintances	M= 211 (14.0)<br>F= 366 (13.1)	Centre-north=89 (4.3)<br>South-islands=490 (21.8)	High (>9)= 319 (13.2)<br>Low (≤9)=260 (13.8)	High >75=503(13.3)<br>Low ≤75=76(14.6)	High >21=175(13.4)<br>Low ≤21=404(13.5)	High >4= 217(13.5)<br>Low ≤4=362(13.5)	Yes=578(16.3)<br>No=1(0.1)
Lack of support from institutions	M= 323 (21.4)<br>F= 581 (20.9)	Centre-north= 882 (43.0)<br>South-islands=22 (1.0)	High (>9)= 510 (21.1)<br>Low (≤9)= 394 (20.9)	High >75=798(21.2)<br>Low ≤75=106(20.3)	High >21=285(21.9)<br>Low ≤21=619(20.7)	High >4= 318(19.8)<br>Low ≤4=586(21.8)	Yes=518(14.6)<br>No=385(52.2)
Costs	M= 700 (46.4)<br>F= 1373 (49.3)	Centre-north= 838 (40.9)<br>South-islands= 1236 (55.0)	High (>9)= 1162 (48.1)<br>Low (≤9)= 912 (48.5)	High >75=1823(48.3)<br>Low ≤75=251(48.1)	High >21=619(47.5)<br>Low ≤21=1455(48.6)	High >4= 760(47.3)<br>Low ≤4=1314(48.9)	Yes=2048(57.6)<br>No=25(3.4)
Lack of time	M= 140 (9.3)<br>F= 195 (7.0)	Centre-north= 77 (3.8)<br>South-islands= 258 (11.5)	High (>9)= 192 (8.0)<br>Low (≤9)= 143 (7.6)	High >75=285(7.6)<br>Low ≤75=50(9.6)	High >21=104(8.0)<br>Low ≤21=231(7.7)	High >4= 130(8.1)<br>Low ≤4=205(7.6)	Yes=15(0.4)<br>No=320(43.4)
Mistrust in effectiveness	M= 133 (8.8)<br>F= 270 (9.7)	Centre-north= 163 (8.0)<br>South-islands= 240 (10.7)	High (>9)= 231 (9.6)<br>Low (≤9)= 172 (9.1)	High >75=364(9.6)<br>Low ≤75=39(7.5)	High >21=121(9.3)<br>Low ≤21=282(9.4)	High >4= 182(11.3)<br>Low ≤4=221(8.2)	Yes=397(11.2)<br>No=6(0.8)

Table S7. Multiple logistic regression of global positive attitudes for pro-environmental behaviors (PAPEB) index. Notable OR_{adj} are in bold.

**Dependent variable: PAPEB** (Global Positive Attitudes for Pro-Environmental Behaviors index)	OR_{crude} (95% CI)	[1]OR_{adj} (95% CI)
Global health risk perception index (GHRP)		
High (>75)	*	*
Low (≤ 75)	3.173 (2.593-3.880)	**2.482 (1.992-3.093)**
Trust in action by institutional (TAI)		
High (> 21)	*	*
Low (≤ 21)	1.449 (1.281-1.639)	**1.192 (1.028-1.383)**
Trust in action by non-institutional subjects (TANI)		
High (> 15)	*	*
Low (≤ 15)	1.942 (1.676-2.252)	**1.513 (1.269-1.804)**
Internet and social as sources of information (yes/no)		
No	*	*
Yes	0.960 (0.837-1.101)	0.975 (0.837-1.137)
Functional health literacy (FHL)		
High (> 9)	*	*
Low (≤ 9)	1.331 (1.169-1.471)	**1.202 (1.057-1.366)**
Gender		
Female	*	*
Male	1.074 (0.953-1.211)	1.086 (0.950-1.240)
Area of residence		
Centre-north	*	*
South-islands	1.065 (0.950-1.195)	0.998 (0.878-1.134)
Global Negative Attitudes (GNA)		
High (> 12)	*	*
Low (≤ 12)	0.788 (0.702-0.884)	0.762 (0.670-0.866)
Adoption of Pro-Environmental Behaviors (APEB)		
High (> 15)	*	*
Low (≤ 15)	2.789 (2.471-3.148)	**2.441 (2.143-2.781)**
Global Support (GS)		
High (> 18)	*	*
Low (≤ 18)	3.473 (3.077-3.919)	**2.777 (2.441-3.159)**

[1]Each odds ratio is adjusted for all other variables in the table. *Reference category.

Table S8. Multiple logistic regression of global adoption of pro-environmental behaviors (APEB) index. Notable OR_{adj} are in bold.

**Dependent variable: APEB (**Global Adoption of Pro-Environmental Behaviors index)	OR_{crude} (95% CI)	[1]OR_{adj} (95% CI)
Global health risk perception index (GHRP)		
High (>75)	*	*
Low (≤75)	1.544 (1.274-1.869)	1.178 (0.958-1.448)
Trust in action by institutional (TAI)		
High (> 21)	*	*
Low (≤ 21)	1.301 (1.148-1.476)	1.058 (0.914-1.224)
Trust in action by non-institutional subjects (TANI)		
High (> 15)	*	*
Low (≤ 15)	1.731 (1.494-2.006)	**1.448 (1.222-1.715)**
Internet and social as sources of information		
No	*	*
Yes	1.235 (1.074-1.421)	**1.225 (1.056-1.421)**
Functional Health Literacy (FHL)		
High (> 9)	*	*
Low (≤ 9)	1.150 (1.022-1.295)	1.062 (0.936-1.205)
Gender		
Female	*	*
Male	0.958 (0.847-1.083)	0.958 (0.841-1.092)
Area of residence		
Centre-north	*	*
South-islands	1.175 (1.044-1.323)	**1.143 (1.008-1.296)**
Global Negative Attitudes (GNA)		
High (> 12)	*	*
Low (≤ 12)	0.805 (0.715-0.907)	**0.826 (0.728-0.938)**
Global Support (GS)		
High (> 18)	*	*
Low (≤ 18)	1.831 (1.625-2.064)	**1.305 (1.145-1.487)**
Positive Attitudes for Pro-Environmental Behaviors (PAPEB)		
High (> 26)	*	*
Low (≤ 26)	2.789 (2.471-3.148)	**2.441 (2.143-2.781).**

[1]Each odds ratio is adjusted for all other variables in the table. *Reference category.
